# Impact of biochar-supported zerovalent iron nanocomposite on the anaerobic digestion of sewage sludge

**DOI:** 10.1007/s11356-019-04479-6

**Published:** 2019-02-13

**Authors:** Min Zhang, Jianhua Li, Yuncai Wang

**Affiliations:** 1 Department of Landscape of Architecture, Center for Ecophronetic Practice Research, College of Architecture and Urban Planning, Tongji University, Shanghai, 200092 China; 2 Key Laboratory of Yangtze River Water Environment of the Ministry of Education, Tongji University, Shanghai, 200092 China

**Keywords:** Sewage sludge, Anaerobic digestion, nZVI-BC composite, Heavy metals, Methane production, Archaeal community

## Abstract

Anaerobic digestion (AD) is an attractive technology for sludge treatment as it stabilizes sludge and produce renewable energy. However, problems such as low organic matter content and high heavy metals level are often encountered which severely limits the effectiveness of AD. In this study, the biochar-supported nanoscale zerovalent iron (nZVI-BC) was synthesized and used as additives during AD of sewage sludge to investigate the enhancement effects for methane production and its impacts on microbial structure at mesophilic temperature. nZVI-BC addition enhanced process stability by improving the generation and degradation of intermediate organic acids, but inhibitory effects were observed at high dosage. The methane content and cumulative methane yields were increased by 29.56% and 115.39%, respectively. Compared with AD without nZVI-BC, the application of nZVI-BC showed positive effect on improvement of metals (Cu, Cd, Ni, Cr, and Zn) stabilization in the digestate. Microbial community analysis illustrated that nZVI-BC addition could significantly increase the Shannon diversity index and Chao1 richness index of archaea, and meanwhile archaea were more diverse in nZVI-BC amended digesters than in control. It was notable that *Methanosaeta* dominated in all the digesters at genera level, while the relative abundance of *hydrogenotrophic* methanogens (*Methanobacterium* and *methanospirillum*) increased 35.39% in nZVI-BC amended digesters compared to the control, resulting in higher methane production. The results will guide development of microbial management methods to enhance the stability of AD process.

## Introduction

Because of the increase in the wastewater treatment capacity, large amounts of sewage sludge as a by-product is produced and stockpiled in environment, which pose a potential environmental risk if not disposed appropriately. Sewage sludge has relative high content of N, P, and other nutrient that is essential for plant growth, and application of sewage sludge in agriculture has been long considered as a sustainable practice owing to the many benefits it provides (Engelhart et al. 2000). Meanwhile, sewage sludge containing hazardous substances such as heavy metals, pathogen, and persistent organic compounds, has potential harm to the environment and can even lead to serious pollution if not properly handled prior to land utilization (Coutand et al. 2008). Related study has demonstrated that heavy metals in untreated sludge are among the most toxic and widespread contaminants because of their high accumulation potential and hardly biodegradable nature (Bruins et al. 2000; Zhao et al. 2016). Therefore, economic and effective approaches are urgently needed prior to landfill or agricultural application due to the high bioavailability of heavy metals in sludge (Donatello and Cheeseman 2013; Wang et al. 2018). Anaerobic digestion is a bio-chemical degradation process of organic materials, with the action of microorganism under anaerobic condition, the biodegradable organics in sludge can be effectively converted to biogas.

Anaerobic digestion, coupled with renewable-energy production in the form of methane, is a complicated biochemical process for waste treatment and utilization (Madsen et al. 2011; Mao et al. 2015). However, problems such as low C/N ratio and operational instability often reduce the digestion efficiency (Appels et al. 2011). This is because anaerobic digestion (AD) process is a multistage, multiphase, and stepwise biodegradation process (hydrolysis, acidogenesis, and acetogenesis and methanogenesis) by a complex community of microorganisms to perform the digestion jobs (Vrieze et al. 2012). Researchers have shown that direct inhibition and indirect inhibition in AD are the major issues, which has a negative effect on microbial activity. Therefore, considerable efforts have been made to improve the activity of microorganism with less problem encountered during the AD process, and the addition of biochar was found to be an effective method (Maroušek et al. 2017). This is because carbonaceous sorbents could be used to adsorb the potential inhibitors from the process due to the abundant pore structures, which can result in improving the activity of the microorganism (Luo et al. 2015). In addition, biochar could also provide a high surface area for the adhesion and growth of microorganism, improve the methane yield, and serve as a soil amendment (Wang and Han 2012).

Biochar, derived from biomass pyrolysis in a closed system with little or no oxygen, has been widely used to remediate contaminated water (Das et al. 2008; Zwieten et al. 2010; Khan et al. 2013; He et al. 2018). Recent studies suggested that biochar as an AD additive could affect microbial activity and community structure, create a surface area to facilitate enzyme immobilization, and reduce nutrients (mainly N and P) losses during AD process (Fagbohungbe et al. 2016). Meanwhile, previous studies have proved that biochar has high adsorption capacities for heavy metals due to its unique properties and cation exchange capacity (Barker 2011; Mohan et al. 2014). However, it has been observed that the pure biochar has the limited adsorption ability due to the limitation of its adsorption mechanisms (Shen et al. 2019). Hence, to overcome the deficiencies of the pristine biochar, modification methods with aim to increase the sorption efficiency have been studied recently (Rajapaksha et al. 2016; Sizmur et al. 2017). Wei et al. (2018a) investigated nitrate removal by biochar-supported nZVI (nZVI-BC) and found 75.0–97.0% of nitrate was removed over a wide pH range 2–12. Bakshi et al. (2018) found that ZVI-biochar complexes, produced by pyrolysis of magnetite and biomass, greatly enhanced As^5+^ removal from contaminated drinking water. Qian et al. (2019) found that rice straw biochar-supported nZVI composite showed better Cr (VI) removal compared to nZVI. Nanoscale zerovalent iron (nZVI), as a reductive material, has recently attracted increased attention in environmental remediation because of good removal capacity of a good array of inorganic and organic hazardous materials (He et al. 2010; Keane 2010). In previous study, it was found that nZVI could reduce the oxidation-reduction potential of the AD system (Liu et al. 2012), enhance methane production, and improve methanogenic activity (Feng et al. 2014). However, nZVI in solution tends to aggregate due to its strong magnetic attraction and the contact area for target pollutants reduced (Cumbal and Greenleaf 2003; TP et al. 2007). With regard to these specific challenges when biochar is utilized as one of the most promising support materials for nZVI due to its high sorption capacity, environmental friendliness and cost-effective (Beesley et al. 2010; Wang et al. 2015b; Zhou et al. 2014). Biochar adsorption and nZVI complement each other, suggesting that biochar to support nZVI particles for remediation of contaminated environment is with the potential of wide applications. The previous studies have indicated that nZVI supported on biochar composite (nZVI-BC) can effectively enhance the immobilization and decrease the bio-availability of heavy metals in soil (Su et al. 2016b, c). Up to date, very few studies have reported to determine the effects of nZVI-BC addition on the stabilization of AD process and immobilization of heavy metals in sewage sludge.

In this work, biochar derived from corn stover was used to support nZVI obtained by the aqueous phase borohydride reduction method. The objectives of this study were as follows: (1) to investigate the performance of nZVI-BC on enhancing methane production and AD process stability of sludge, (2) to evaluate the effectiveness of nZVI-BC addition on the changes in heavy metals speciation and bioavailability in the end products of AD, and (3) to study the microbial community structures and the diversity of archaea changes in the different reactors.

## Materials and methods

### Sewage sludge and biochar

The raw sludge samples were collected from a local municipal wastewater treatment plant in Hefei, Anhui Province, China. The raw sludge in the study was characterized and it contained 10.9 ± 0.27% total solids (TS), 53.7 ± 0.33% volatile solids (VS) of TS, 4.5 ± 0.17 C/N ratio, and 6.6 ± 0.19 pH. The total contents of the target heavy metals were 132.57 ± 3.02 mg kg^−1^ Cu, 179.62 ± 6.21 mg kg^−1^ Cr, 92.91 ± 2.79 mg kg^−1^ Ni, 2.72 ± 0.09 mg kg^−1^ Cd, and 1963.79 ± 6.59 mg kg^−1^ Zn. The source of inoculum was a sludge obtained from a long-term continuous lab-scale anaerobic digester in the National Engineering Research Center for Urban Pollution (NERCUPC) at Tongji University. The main characteristics of the inoculated sludge were as follows: TS, 9.6 ± 0.22% (*w*/*w*) and VS were 49.1 ± 1.3% of TS. After collecting the sludge samples, part of it was dried, ground, and filtered through 0.22-μm filter membranes for physicochemical characterization. The rest of the sludge was freeze-dried and used for AD process. The biochar used in the study was derived from pyrolysis of corn stover. About 600 g of the dried corn stover were turned into biochar through continuously high-temperature pyrolysis under constant N_2_ gas production.

### Synthesis of biochar-supported nZVI

Corn stover was oven dried at 85 °C and pyrolyzed in a sealed tube furnace to produce pristine biochar at 550 °C for 120 min under constant N_2_ gas production. The obtained biochar was successively rinsed with 1 M HCl, tap water, and distilled water to remove the suspend ash, and then oven dried overnight at 80 °C. Biochar-supported nZVI composites were synthesized according to methods proposed by Jabeen et al. (2013) with some moderate modification. The reduction reaction is carried out in the following equations:

1 $$ 4{\mathrm{Fe}}^{3+}+3{\mathrm{BH}}_4^{-}+9{\mathrm{H}}_2\mathrm{O}\to 4{\mathrm{Fe}}^0+3{\mathrm{H}}_2{\mathrm{BO}}_3^{-}+6{\mathrm{H}}_2+12{\mathrm{H}}^{+} $$

Biochar-supported nZVI composite was then prepared by liquid-phase reduction process (Dong et al. 2017). Briefly, 2.5 g prepared corn straw biochar was first dispersed in 50 mL 1 M FeCl_3_ solution for 12 h with stirring under room temperature. Subsequently, the mixture was transferred into a three-neck flask and then the N_2_ was purged into the solution under continuous stirring for 1 h to exclude the dissolved O_2_. Then 0.12 M borohydride (NaBH_4_) solution was added dropwise to the above solution followed by 30 min of stirring and continuous N_2_ purging. The suspension was vacuum filtered and then washed several times with deionized water followed by ethanol. After vacuum dried at 60 °C for 12 h, the biochar-supported nZVI composite was finally prepared and then stored in sealing containers at 4 °C prior to use.

### Characterization of BC and nZVI-BC

The total carbon, hydrogen, and nitrogen contents of BC and nZVI-BC composite were determined using an Elemental analyzer (Elementar Analysensysteme Gmbh, Hanau, Germany). The Fe content in BC and nZVI-BC was determined by inductively coupled plasma atomic emission spectrometry (ICP-AES) (Perkin Elmer Optima 2100 DV ICP-AES). Ash contents of the materials were defined as the mass remaining after pyrolysis at 750 °C in a crucible until a constant weight was obtained. Brunauer-Emmett-Teller (BET)-specific surface area was measured using ASAP2020 surface area and porosity analyzer (Micromeritics, Norcross, USA). Scanning electron microscope (SEM, Hitachi 4700 microscope, Hitachi) was used to determine the morphology of the samples. The surface chemistry analysis of the composite was conducted using X-ray photoelectron spectroscopy (XPS, ESCALAB 250Xi). Transmission electron microscopy (TEM) analysis was carried out using a JEM-2100F (JEOL, 200 kV) to observe the microstructure of the materials.

### Anaerobic digestion experimentation

The batch experiments were performed in 500 mL serum bottles at mesophilic temperature (37 ± 1) °C with a working volume of 450 mL, and each set of experimental condition was conducted in triplicate. The inoculum to substrate ratio was set between 0.31 and 0.33 based on the volatile solid. Seven sets of batch experiments were set up and the design is as follows: (R0):150 g sludge without BC or nZVI-BC; (R1): 150 g sludge with 0.75 g BC (0.064 g/g dry matter); (R2): 150 g sludge with 0.75 g nZVI-BC (0.064 g/g dry matter); (R3): 150 g sludge with 1.50 g nZVI-BC (0.13 g/g dry matter); (R4): 150 g sludge with 2.50 g nZVI-BC (0.21 g/g dry matter); (R5): 150 g sludge with 4.00 g nZVI-BC (0.34 g/g dry matter); and (R6): 150 g sludge with 5.00 g nZVI-BC (0.43 g/g dry matter). Sludge samples were well homogenized and diluted to 10% of total solid and then sonicated for 20 min. After feeding the reactors, each of the bottle was tightly sealed with rubber plugs and then secured with aluminum after being flushed with high-purity nitrogen gas (> 99.99%) (Koch et al. 2015). Thereafter, all batch reactors were incubated in a thermostatic shaker at (37 ± 1) °C and 170 rpm until no significant methane production was observed, within approximately 34 days.

### Metal contents and sequential fractionation

The total concentrations of heavy metals were measured by digesting the dry sludge samples with aqua regia for 180-min reaction time (Dong et al. 2013). Then the extracts were filtered through 0.22 μm filter membranes before analyzing the total metal content on an ICP-AES instrument (Perkin Elmer Optima 2100 DV ICP-AES). The modified sequential extraction procedure as reported by Aikpokpodion et al. (2013) was implemented to analyze the speciation of Cu, Cd, Ni, Cr, and Zn in sludge samples with six steps, which can be presented in sequence: step one, water-soluble fraction (F1); step two, exchangeable fraction (F2); step three, carbonate-bound fraction (F3); step four, Fe-Mn oxides-bound fraction (F4); step five, organic and sulfide-bound fraction (F5); and step six, residual fraction (F6). The details of the modified sequential extraction procedures were reported in our previous study (Zhang et al. 2016). The mobility factors (MFs) of heavy metals was calculated as the ration between the sum of the water-soluble, exchangeable, and carbonate-bound fractions (F1, F2, and F3) and the total concentration.

### Analytical methods for chemical index

The biogas production and methane content were measured every day. The analytical methods for TS, VS, alkalinity, ammonia nitrogen, TCOD, and SCOD were determined following the standard methods for the examination of water and wastewater (APHA 1998). The pH vales were measured using a pH meter (pHS-2F, Shanghai Precision and Scientific Instrument Co. Ltd., Shanghai, China). Gas and liquid sludge samples were collected periodically for analysis until biogas production ceased. Approximately 8 mL of biogas samples was withdrawn from each of the reactors and stored in an air bag, and the methane content in biogas were determined by gas chromatograph (GC, Agilent Technologies 6890N, CA, USA) equipped with a thermal conductivity detector (TCD) using helium gas as a carrier gas. The volatile fatty acids (VFA) were detected with a gas chromatograph (GC, Agilent Technologies 6890N, CA, USA) equipped with a flam ionization detector (FID). All the measurements in the study were carried out in triplicates and the average values were reported.

### Archaeal community analysis

The archaea communities in reactors before and after nZVI-BC addition were investigated by Illumina high-throughput sequencing. Sludge samples were collected from the reactors after anaerobic digestion for 34 days. About 7 mL of sludge samples were immediately freezed at − 20 °C for microbial analysis. DNA was extracted using the MoBio Ultra Clean™ soil DNA isolation kit (MoBio Laboratories, Inc., Carlsbad, CA) according to the manufacturer’s instruction. The DNA was quantified by 2% agarose gel electrophoresis and its purity was determined by measuring the 260/280 nm absorbance ratio (Chen et al. 2015). DNA from the sludge was amplified by PCR using archaea primer sets 349F (CCCTACACG-ACGCTCTTCCGATCTN (barcode) GYGCASCAGKCGMGAAW) and 806R (GACTGGAGTTCCTTGGCACCCGAGAATTCCAGGACTACVSGGGTATCTAAT) for PCR amplification (Su et al. 2016a; Zhang et al. 2018). Amplifications were carried out as follows: initial denaturation at 94 °C for 3 min, followed by 20 cycles of denaturation at 94 °C for 20 s, primer annealing at 55 °C for 20 s, and extension at 72 °C for 30 s and a final extension at 72°Cfor 5 min. After amplification, sludge samples were sent out for pyrosequencing on the Illumina MiSeq platform (Shanghai Sangon Technology Co., Ltd). Rarefaction curve and alpha diversity statistics (the Shannon index, the Simpson index, and the Chao1 richness) were analyzed for each sludge samples (Shu et al. 2015).

### Statistical analysis

In this work, calculation of arithmetical mean and standard deviations were performed using Microsoft Excel 2016. Statistical analysis was carried out using the software version SPSS 13.0 for windows (SPSS, Chicago, IL). Duncan’s new multiple range test (DMRT) was employed to assess the differences between the treatment means. One-way AVONA analysis was implemented to assess the statistical significance of differences between the mean results of different treatments. The level of significance was set to *p* ≤ 0.05.

## Results and discussion

### Properties of BC and nZVI-BC

Table 1 shows the elemental analysis and surface area analysis of BC and nZVI-BC. Elemental compositions of nZVI-BC had lower carbon content (85.9% for BC and 71.9% for nZVI-BC) and hydrogen content (1.72% for BC and 0.62% for nZVI-BC) than BC. Compared to BC, nZVI-BC had higher O/C ratio (0.07 for BC, 0.15 for BC-nZVI), suggesting that nZVI-BC composite contained more oxygen functional groups and had better adsorption ability (Yang et al. 2018). Bulk Fe content in nZVI-BC composite was about 136.25 times greater than that in biochar, indicating that iron was successfully impregnated in BC. In comparison, the average pore width of nZVI-BC increased significantly as shown in Table 1. The possible reason may be that through chemical modification, the volatile was released from the biochar matrix as well as the incorporation of nanoparticles which facilitated the enlargement of the existing pores, as reported by Zhu et al. (2015). The specific surface area of BC and nZVI-BC were 61.9 m^2^/g and 16.7 m^2^/g respectively. The decreased specific surface area of nZVI-BC was attributed to the reason that the pore structure of biochar was completely blocked by nZVI particles (Zhu et al. 2019). It is also possible that the pore structure of biochar was destroyed or collapsed by chemical reactions during chemical modification (Neeli and Ramsurn 2018).

**Table 1:** Physical and chemical characteristics of BC and nZVI-BC

Samples	C (%)	H (%)	N (%)	O (%)	Fe (%)	S_BET_ (m^2^/g)	Pore width (nm)
BC	85.9	1.72	0.91	6.02	0.07	61.9	21.3
nZVI-BC	71.9	0.62	0.59	10.71	10.9	16.7	59.2

**Fig. 1:**
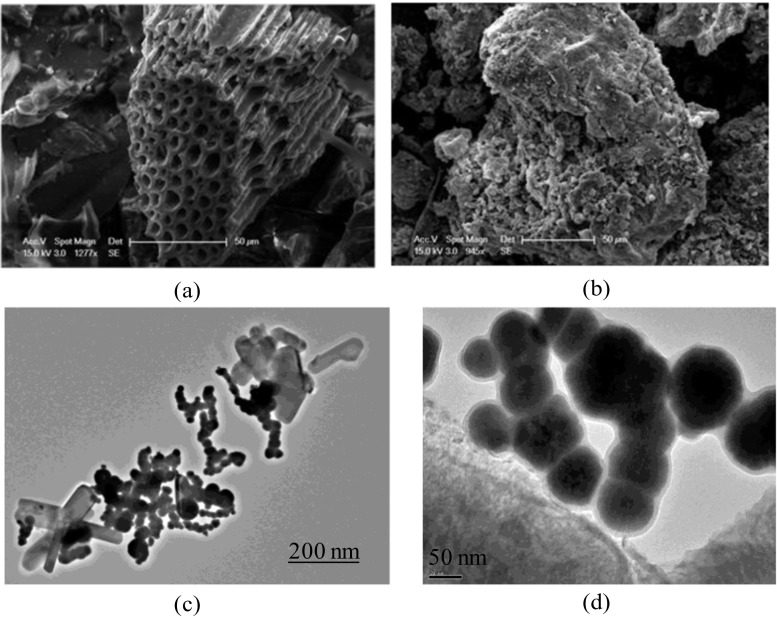
SEM of the biochar (**a**) and nZVI-BC composite (**b**). TEM images of nZVI-BC (**c**, **d**)

The surface morphology and microstructure of BC and nZVI-BC were examined using SEM and TEM. SEM images (Fig. 1a, b) showed that both biochar and nZVI-BC consist of irregularly shaped particles with rough surface and abundant porous structure. Obviously, nZVI-BC was rougher than BC and had more micropores. The TEM images (Fig. 1 c, d) showed that the nanoparticles imbedded in biochar matrix were spherical and easily agglomerated, whereas the carbon support can effectively inhibit agglomeration.

**Fig. 2:**
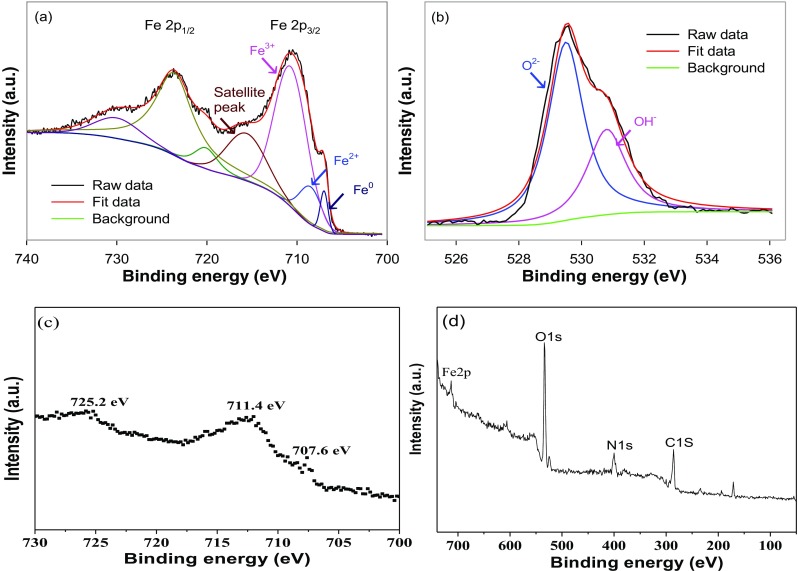
XPS spectra of nZVI-BC. Spectra were obtained by plotting counts against binding energy for Fe 2p (**a**, **c**), O1s (**b**), and overall survey (**d**) in a wide scan

XPS was performed to investigate the surface composition and chemical states of nZVI-BC composite. The XPS survey spectrum (Fig. 2a, b) conforms the presence of C, N, O, and Fe elements in the composite. The two intense peaks at 711.4 eV and 725.2 eV in the high-resolution Fe 2p XPS spectrum correspond to the 2p3/2 binding energies of Fe/Fe_2_O_3_/Fe_3_O_4_/Fe (OH) O and 2p1/2 binding energies of FeOOH/Fe_2_O_3_ composites, respectively (Tian et al. 2016). And the peaks at 707.6 eV indicated the presence of nZVI (Yoon et al. 2011). The intense peaks at 530.7 eV (O 1S) showed that the presence of metal oxides on account of the oxidation of surface nZVI particles to Fe_2_O_3_/Fe_3_O_4_/Fe (Tian et al. 2010; Qiu et al. 2011).

### Effect of nZVI-BC addition on methane production from anaerobic digestion of sewage sludge

**Fig. 3:**
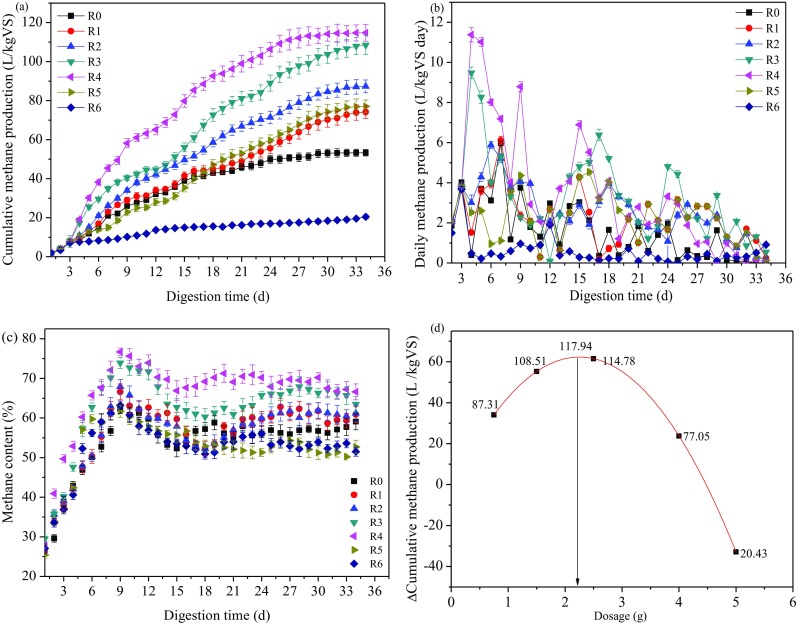
**a** Variation of cumulative methane production and **b** daily methane production. **c** Variation of methane content during anaerobic digestion. **d** Relationship between △cumulative methane production and dosage of nZVI-BC addition

The experimental reactors run in batch mode at 37 °C for 34 days. As key parameters for evaluating the anaerobic digestion performance, the cumulative methane production, daily methane yield, and the methane content of biogas are shown in Fig. 3. The cumulative methane production after 34 days digestion reached 53.29 ± 1.66, 74.13 ± 3.26, 87.31 ± 3.29, 108.51 ± 4.72, and 114.78 ± 4.35 L kg^−1^ VS for digesters with 0, 0.75 g BC, 0.75 g nZVI-BC, and 1.50 g nZVI-BC treatment, respectively (Fig. 3a). At mesophilic temperature, compared to the control, the BC and nZVI-BC amendment raised the cumulative methane yield by 39.11% and 63.84% for the digesters with 0.75 g BC and 0.75 g nZVI-BC treatment, respectively. The highest cumulative methane production was observed in the reactors with 2.50 g nZVI-BC treatment, 115.39% higher than the control and 31.46% higher than that in the reactors with 0.75 g nZVI-BC treatment. However, the digesters with 5.00 g nZVi-BC exhibited the lowest cumulative methane yield, which was 61.66% lower than the control. And the result was in accordance with Su et al. (2013), who found that the methane concentration in produced biogas and methane yield increased by 5.1–13.2% and 40.4% when nZVI was added in the anaerobic digestion reactors after 17 days of digestion, respectively. Results showed that right amount of nZVI-BC could promote the sludge cells dissolution of organics and enhance the adaption and activity of methanobacteria (Wei et al. 2018b; Zhang et al. 2015) while the excessive dosage of nZVI can inactive bacteria by causing serious damage to the cell membranes and methane yield decrease (Xie et al. 2017). The higher methane production compared to the control might be caused by four main reasons. First, biochar is of porous structure with large surface area, which has used as an adsorbent to remove metabolite such as acetic acid and hydrogen iron required for methane production (Bagreev et al. 2001). Second, nZVI can be degraded to form iron hydroxide and iron oxides minerals surrounding the nZVI core, and the reduction/oxidation cycle of Fe^3+^/Fe^2+^ and zero iron in the core accelerated the electron flow from the oxidation of acetate to *hydrogenotrophic* methanogenesis (Jiang et al. 2013). Third, nZVI can be oxidized to Fe^2+^ under anoxic condition (Fe^0^ + 2H^+^ → Fe^2+^ + H_2_), nZVI and produced H_2_ could serve as electron donor for reduction of CO_2_ to CH_4_ which resulted in the improvement of methane production (Suanon et al. 2017; Jiang et al. 2013) (As shown in Eqs. (2) and (3)). nZVI consumed hydrogen ions and meanwhile supplied electrons, which were used by methanogens to enhance activity and metabolic rates. Finally, Fe contained in the nZVI-BC composite is known as essential micronutrients for enzyme immobilization as well as supporting metabolisms during AD process (Choong et al. 2016). The presence of biochar was efficient in decreasing the aggregation of nZVI particles so that its activity was relatively higher compared with the bare nZVI (Gong et al. 2017). However, the methanogenesis inhibition through the sludge anaerobic digestion process is attributed to the disruption of cell membrane that massive dosage of nZVI particles caused (Yang et al. 2013).

2 $$ {\mathrm{CO}}_2+2{\mathrm{H}}_2\to {\mathrm{CH}}_4+2{\mathrm{H}}_2\mathrm{O} $$

3 $$ \kern3.00em 4{\mathrm{Fe}}^0+8{\mathrm{H}}^{+}+{\mathrm{CO}}_2\to 4{\mathrm{Fe}}^{2+}+{\mathrm{CH}}_4+2{\mathrm{H}}_2\mathrm{O} $$

Figure 3b showed the daily methane yield in digesters and it was obvious that there were significant differences (*p* < 0.05) between different treatment groups. The methane production increased rapidly in the start-up stage of the AD process (< 5–7 days), while the amount of methane production rose up little after digestion for 20 days. This is because the initial methane production peak could correspond to the decomposition of the available soluble organic substrates in the sludge; afterwards, the methanogenic activities were inhibited due to the substantial accumulation of VFA and available organic substrates were being metabolized (Xi et al. 2014). The maximum daily methane production was observed in the digesters with 2.50 g nZVI-BC treatment, which yields 11.38 L kg^−1^ VS/day on day 5, while the other additives were unable to yield as high as that.

For mesophilic operation, methane content in the AD process changed along with the dosage of nZVI-BC addition. As shown in Fig. 3c, results showed that methane content of all digesters were very low in the early stages of AD (about 25.4–37.2%). It could be concluded that the microbial activity was partially inhibited and was still in adjustment. After 6 days digestion, methane content increased sharply which could be due to the microorganism adapted to the new environment and microbial activity recovered. The highest methane content was achieved in the reactors with 2.50 g nZVI-BC treatment, reaching 76.7% on day 9, which was 18.77% higher than the control (62.3%). However, the methane content was lower in the reactors with 5.00 g nZVI-BC treatment, no more than 65% during the whole AD process. The previous studies showed that AD process reached into equilibrium when the methane content in system was superior or equal to 60% (Yuan et al. 2016). The methane content from all the nZVI-BC amended digesters and the control dramatically dropped after day 27. Dearman and Bentham (2007) had reported that decreases in methane content were related to the limited available carbon-base nutrients for producing methane.

As depicted in Fig. 3d, a strong correlation (*R*^2^ = 0.9935) between the dosage of nZVI-BC and the increment of cumulative methane production (△cumulative methane production) was found under the test conditions in the experiment (as shown in Eq. (2)).

The maximum cumulative methane production (117.94 L/kg VS) was obtained when 2.25 g nZVI-BC was added to the mesophilic AD system. That is, there is a strong and positive relevance between the cumulative methane yields and the dosage of nZVI-BC below 2.25 g, while a falling limb occurred if surpassed. Besides, it was assumed that when the dosage of nZVI-BC increased to 4.49 g, the gas production stopped, and the AD process was completely inhibited. Therefore, in the experiment, the optimal doses of nZVI-BC were 2.25 g, which is beneficial to enhance methane production due to its ability to promote biofilm formation and improve the microorganism activity in the sludge (Mumme et al. 2014; Torri and Fabbri 2014).

### Impact of nZVI-BC addition on digesters performance

**Fig. 4:**
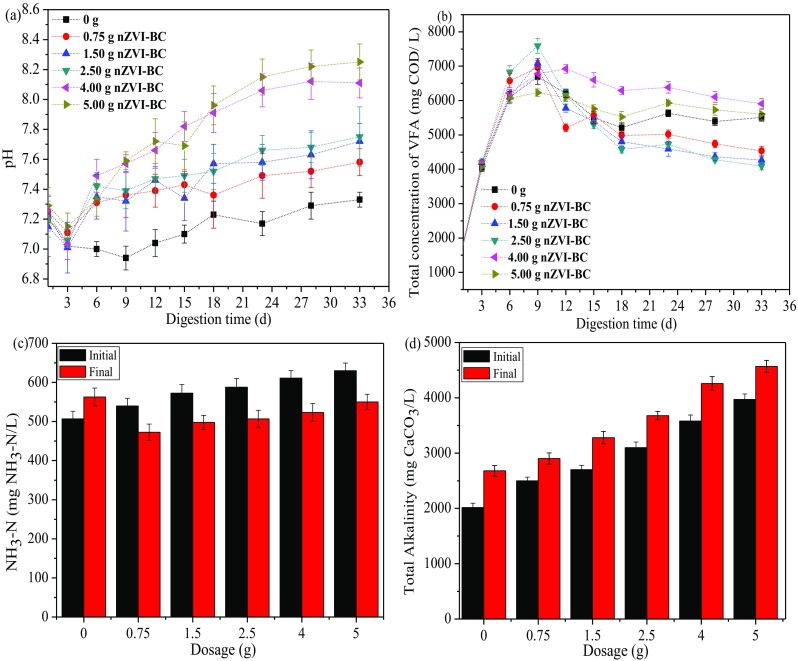
Digester environment before and after mesophilic anaerobic digestion: **a** variation of pH, **b** variations of total volatile acid, **c** variation of NH_3_-N, and **d** variation of total alkalinity

Figure 4 shows the characteristics of the system including pH, VFA, NH_3_-N, and alkalinity after 34 days of AD, and the values of these parameters could well manifest the stability of the AD system (Ying et al. 2015). The digester pH is an important parameter which influences the microbial activity and AD process stability (Watling et al. 2013). Previous research showed that the most favorable pH for methanogenic activity was in the range of 6.6–7.8 (Shi et al. 2014). In the study, the pH changes had four stages, and the initial pH was adjusted to 7.2 ± 0.2 in all digesters. As shown in Fig. 4a, the pH value decreased at the beginning of the digestion due to the accumulation of VFA. After dosing different dosages of nZVI-BC, a turning point was obtained due to the alkaline of the nZVI-BC composite. Afterwards, the drastically decrease of pH in the digesters with or without nZVI-BC addition might be due to the consumption of the VFA accumulated in early stage of AD process (Ahn et al. 2010). After 23 days, the pH value variation trended to be steady. Moreover, the digester with 2.50 g nZVI-BC treatment maintained the pH at the level of 7.5–7.6, which could be attributed to the buffering role that nZVI played by the dissociation of Fe^0+^$\left(4{\mathrm{F}\mathrm{e}}^0+{\mathrm{CO}}_2+8{\mathrm{H}}^{+}\to 4{\mathrm{F}\mathrm{e}}^{2+}+{\mathrm{CH}}_4+2{\mathrm{H}}_2\mathrm{O};{\mathrm{F}}_{\mathrm{e}}+2{\mathrm{H}}_2\mathrm{O}\to {\mathrm{F}}_{\mathrm{e}}^{2+}+{\mathrm{H}}_2+2{\mathrm{OH}}^{-}\right)$(Li et al. 2007; Ganzoury and Allam 2015).

To evaluate the AD process stability, the VFA variation was measured during AD process in different treatment digesters. As shown in Fig. 4b, TVFA in anaerobic fermentation kept increasing during the initial 9 days, due to the decomposition of organics in sludge and lower activity of anaerobic microbe. The group with 2.50 g nZVI-BC treatment had the largest TVFA concentration at the acid production stage, up to (7591.1 ± 216.92) mg COD/L; and then kept the TVFA concentration at the level of (4579.2–4089.1) mg COD/L at the methane production stage. The cultures with nZVI-BC addition (R5 and R6 excepted) degraded VFA faster than the control during the first 18 days of the experiment, suggesting that the moderate amount of nZVI-BC is beneficial to improve the activity of methanogens and enhanced the degradation of organics. As it was well-known that the conversion of propionate to acetate is unfavorable in thermodynamics (as shown in Eqs. (4) and (5)) (Meng et al. 2013), the accumulation of propionate in digesters has a negative effect on activities of methanogens. The addition of nZVI-BC effectively increased the total VFA yield and optimized the thermodynamically beneficial condition to avoid propionic acid-type fermentation, which would benefit methane production (Wei et al. 2018b). Besides, we found that more H_2_ was likely produced in the conversion of propionate to acetate, which served as direct substrate and utilized by the hydrogenotrophic methanogens. The above results indicated that the addition of nZVI-BC enhanced the methanogenic activity and accelerated the conversion of VFA.

4 $$ \mathrm{C}{\mathrm{H}}_3\mathrm{C}{\mathrm{H}}_2\mathrm{CO}{\mathrm{O}}^{-}+3{\mathrm{H}}_2\mathrm{O}\to \mathrm{C}{\mathrm{H}}_3\mathrm{C}\mathrm{O}{\mathrm{O}}^{-}+\mathrm{HC}{\mathrm{O}}_3^{-}+{\mathrm{H}}^{+}+3{\mathrm{H}}_2 $$

             ∆G = 76.1 (kJ/mol)

5 $$ \mathrm{C}{\mathrm{H}}_3\mathrm{C}{\mathrm{H}}_2\mathrm{C}{\mathrm{H}}_2\mathrm{C}\mathrm{O}{\mathrm{O}}^{-}+2{\mathrm{H}}_2\mathrm{O}\to 2\mathrm{C}{\mathrm{H}}_3\mathrm{C}\mathrm{O}{\mathrm{O}}^{-}+{\mathrm{H}}^{+}+2{\mathrm{H}}_2 $$

∆G = 48.1 (kJ/mol).

Researchers have shown that free ammonia is more toxic than ammonium nitrogen due to its ability to penetrate the cell membrane (Chen et al. 2008). Figure 4c showed that the contents of NH_3_-N in the six treatment all increased after anaerobic digestion except the control. Previous research indicated that the inhibiting threshold of NH_3_-N concentration is from 1700 to 2500 mg/L (Hashimoto 1986), and all the digesters in the experiment were below the value. Specially, the NH_3_-N concentration after AD increased with the increase of nZVI-BC application amount, which could be attributed to the higher pH with nZVI-BC amendment is beneficial to shift the NH_3_/NH_4_^+^ dissociation equilibrium toward the NH_3_ formation (NH_4_ + OH^−^ → NH_3_ + H_2_O). During mesophilic AD, the NH_3_-N concentration increased by 11.11% in the control digester, whereas it decreased 12.45–14.41% after AD in nZVI-BC digesters compared to the control. The results indicated that the feasibility of using nZVI-BC to alleviate NH_3_ inhibition. Huang et al. (2015) reported that the released iron ions (Fe^3+^/Fe^2+^) in the fermentation liquor were supposed to form mineral precipitates with NH^4+^, probably contributed greatly to the decreased ammonia concentration and enhanced the AD process stability. Meanwhile, nZVI-BC addition might alleviate NH_3_ inhibition with a high specific surface area capable of adsorbing ammonia during AD process (Shen et al. 2016).

The total alkalinity concentration is a key index controlling the stability of AD and was measured at the beginning and end of the study under the test condition. The total alkalinity concentrations of all nZVI-BC amended digesters increased and were all significantly higher than those of the control after AD (Fig. 4d), which is mainly due to the metal cations (Na^+^, K^+^, and Ca^2+^) release from the surface of the biochar as well as NH_3_ can react with CO_2_ to generate HCO_3_^−^/CO_3_^2−^ buffer (Shen et al. 2016). At mesophilic temperature, compared to the control, the nZVI-BC amendment raised the total alkalinity concentration by 8.34%, 22.39%, 37.25%, 58.88%, and 70.49% of digesters with 0.75 g, 1.50 g, 2.50 g, 4.00 g, and 5.00 g nZVI-BC treatment, respectively. Higher alkalinity indicates stronger buffer ability and more stability of AD system. Furthermore, VFA/alkalinity ratio is another criterion for judging the stability of AD system, and the value of the ratio below 0.4 indicates that the digestion process could operate stably (Chen et al. 2017). Values of VFA/alkalinity in the experiment were far below 0.4 in the reactors with 2.50 g nZVI-BC treatment, while the values were 0.42 and 0.46 for the reactors with 4.00 g and 5.00 g nZVI-BC treatment, respectively. The improved total alkalinity concentration can enhance buffering capacity and prevent pH drop resulting from the organic acid obtained in AD process, indicating the strong stability for the AD process.

### Heavy metal immobilization by nZVI-BC

**Fig. 5:**
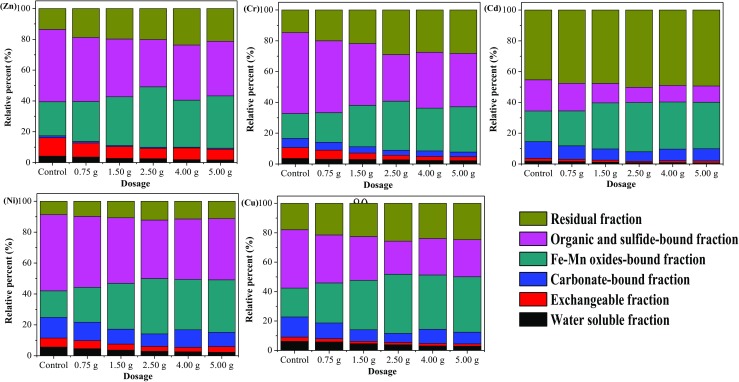
Heavy metals speciation distributions in different digesters of sewage sludge

The species distribution of heavy metals is critical in the determination of toxicity and migration of heavy metals and directly affects the possibility of sludge application (Wang et al. 2007). Heavy metals usually are divided into bioavailable (F1 + F2 + F3), potentially bioavailable (F4 + F5), and non-bioavailable (F6) fractions depending on their mobility and eco-toxicity in environment (Wang et al. 2015a, b). The modified Tessier sequential extraction procedure was used in the experiment to determine the chemical speciation of Cr, Cd, Ni, Cu, and Zn in the digesters after AD. Figure 5 shows the speciation distribution of heavy metals in six fractions. It was observed that the addition of nZVI-BC had notably affected the speciation distribution of the target heavy metals in sewage sludge after AD. The result shows that less than 7.0% of the target heavy metals were released into the water solution, and the water-soluble heavy metal contents gradually decreased with the increase of nZVI-BC application amount. As can be seen, porous structure of biochar could adsorb or react with heavy metals in sludge to form stable metals complex owing to the functional groups on the biochar’s surface (Puga et al. 2015). Meanwhile, studies showed that nZVI was an effective reducing agent and can be used to remove a variety of heavy metals through chemical reduction (Eglal and Ramamurthy 2015; Wang et al. 2017). The strong synergistic effects between the surface properties of biochar and zero valent iron greatly enhanced the adsorption efficiency of heavy metals in sludge.

Comparisons of each digesters showed that different mixture ratios have a significant effect on the fraction distribution at the mesophilic conditions. During the AD process, percentages of bioavailable metals in the F1, F2, and F3 fractions varied with an average decrease of 54.09% Cr, 32.35% Ni, 47.37% Cd, 48.46% Cu, and 35.89% Zn in digesters with 2.50 g nZVI-BC treatment compared to the control. The variation about the five target heavy metals were not the same, the content of available Cr decreased the most, followed by Cu, Cd, Ni, and Zn. Shang et al. (2017) also stated that the nZVI-BC composite was an effective reagent for treating wastewater contaminated with Cr (VI). The groups with 2.50 g nZVI-BC treatment had the best effect on heavy metal immobilization, followed by the digesters with 1.50 g and 4.00 g nZVI-BC treatment. However, greater amounts of amended nZVI-BC do not necessarily lead to enhance the stability of the target heavy metals, and the result was consistent with Diao et al. (2018).

Therefore, based on the data, it can be concluded that nZVI-BC can effectively improve the stability of heavy metals in sludge which attribute to a synergistic effect on the adsorption coupled reduction over the nZVI-BC composite. Along with its oxygen-containing functional groups on biochar surface, nZVI-BC provided more reactive sites for heavy metals to generate stable metal complexes. The main mechanism about adsorption of metals by biochar mainly occurs though electrostatic attraction, iron-exchange, and precipitation onto the surface of the composite (Fagbohungbe et al. 2016). Moreover, the presence of biochar was successfully utilized to decrease the aggregation of nZVI particles to increase their reactivity in environmental application (Table 2).

**Table 2:** The sum of water-soluble, carbonate-bound, and Fe-Mn oxide-bound fractions of heavy metal in solid phase after anaerobic digestion

nZVI-BC	Cr (mg/kg)	Ni (mg/kg)	Cd (mg/kg)	Cu (mg/kg)	Zn (mg/kg)
Control	35.35 ± 1.2^a^	40.59 ± 1.1^b^	0.38 ± 0.01	38.87 ± 1.28	366.37 ± 11.2
0.75 g	28.25 ± 1.0	37.76 ± 1.2	0.32 ± 0.01	31.94 ± 1.21	339.59 ± 11.29
1.50 g	22.96 ± 0.92	31.75 ± 0.96	0.25 ± 0.01	24.52 ± 0.79	244.49 ± 11.72
2.50 g	16.23 ± 0.76	27.46 ± 0.69	0.20 ± 0.01	20.04 ± 0.89	234.88 ± 9.02
4.00 g	23.16 ± 0.95	30.43 ± 1.19	0.27 ± 0.01	29.19 ± 1.23	207.50 ± 8.71
5.00 g	21.17 ± 0.91	27.51 ± 0.79	0.28 ± 0.01	25.14 ± 0.72	193.60 ± 7.79

^a^Values represent the means ± standard deviation (SD)

^b^The date are expressed as the mean value ± standard deviation (*n* = 3)

### Response of the archaeal community to biochar addition

After filtering low quality and trimming the adapters, primers, and barcodes, there were 263,683 (S1), 28,904 (S2), 29,381 (S3), 28,576 (S4), 23,454 (S5), and 26,696 (S6) high-quality sequence reads in the digesters with the control; 0.75 g, 1.50 g, 2.50 g, 4.00 g, 5.00 g nZVI-BC treatment, separately; and a total of 132 archaeal OTUs grouped into 15 genera. Among them, 99.9% of the 132 OTUs were classified in orders Methanosarcinales, Methanomicrobiales, and Methanobacteriales. *Methrix*, *methanothrix*, *methanobacterium*, *methanospirillum*, and *methanosarcina* were the most abundant archaea at genus level (99.1 ± 11.2%). The other genera only accounted for very little percent in all archaeal reads. It was implied that nZVI-BC addition did not change the species but the abundance of archaea, which was consistent with the previous studies (Kong et al. 2018). To identify the microbial community changes in digesters after addition of nZVI-BC, the richness and diversity of species were analyzed using Illumina high throughput sequencing. A higher Shannon index and lower Simpson index represents better richness of the microbial population and the larger number of species (Zhang et al. 2018).

As shown in Table 3, for the bacteria community after nZVI-BC addition, the Shannon index increased from 1.70 (S1) to 2.00 (S4) and the Simpson index decreased from 0.29 (S1) to 0.23 (S4). Shannon diversity index is usually used to evaluate the community species of the microbial community. So, archaeal community in S4 samples were more diverse than the other samples due to the higher Shannon index and lower Simpson index. Chao1 estimators suggested that the S4 samples possessed the highest microbial richness among the six different treatments, while the S6 samples shown the lowest. These results revealed that moderate dosage of nZVI-BC addition could increase the diversity of microbial community.

**Table 3:** Microbial community diversity analysis of bacteria and archaea of nZVI-BC amended and control groups

Sample	OTU num	Shannon index	ACE index	Chao1 index	Simpson
S1	176	1.70	259.69	253.08	0.27
S2	199	1.70	262.72	258.52	0.29
S3	193	1.88	282.30	272.16	0.25
S4	215	2.00	341.96	295.78	0.23
S5	216	1.90	307.90	277.75	0.24
S6	180	1.70	289.34	244.02	0.27

**Fig. 6:**
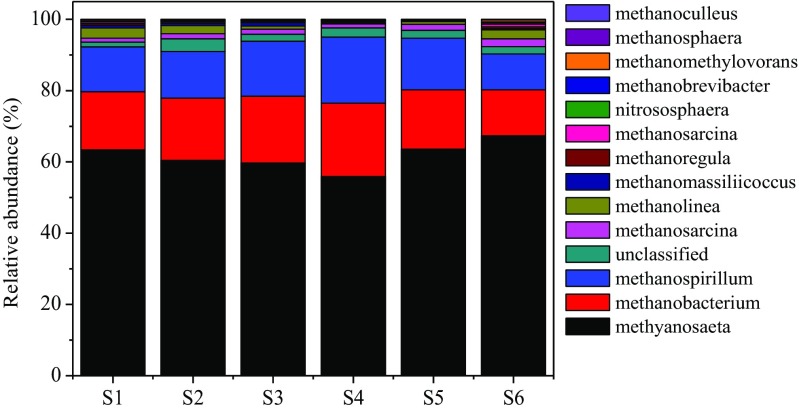
The relative abundance of archaeal community in different treatments at gene level

In order to identify the phylogenetic diversity of archaeal communities in the six different treatment digesters, the relative abundance at genus levels was studied in the research. For the archaea community, as shown in Fig. 6, *methanosaeta*, *methanobacterium*, and *methanospirillum* were the three most abundant archaea at genus level in six reactors. And the most dominant genus in the six digesters was *methanosaeta* species, accounting for 63.4%, 60.45%, 59.7%, and 55.93% in the S1, S2, S3, and S4 samples, respectively. *Methanosaeta* was strict anaerobe and could only use acetate to produce methane (Yuan et al. 2016). *Methanobacterium* and *methanospirillum* are typical H_2_-utilizing methanogen which could produce methane by reducing CO_2_ with H_2_ and formic acid (Garcia et al. 2000; Ye et al. 2017), and their relative abundance in nZVI-BC addition reactors were higher than that in the control. In contrast, the relative abundance of *methanosaeta* decreased from 63.4% in S1 to 55.93% in S4, indicating that the anaerobic and high-solid environment would weaken the competitive advantages of aceticlastic methanogens, and more *hydrogenotrophic* methanogens occurred in nZVI-BC implemented digesters. The above analysis results are in accord with the research by Liu et al. (2016). Previous studies reported that *hydrogenotrophic* methanogens were more robust and therefore, more abundant than aceticlastic methanogens during sludge anaerobic digestion process (Fan et al. 2013). The obtained results implied that the nZVI-BC treatment could effectively enhanced sludge anaerobic digestion led to a community shift from the initial sludge to acclimated communities of the anaerobic digestion system, with the enrichment of specialized methanogens. Ye et al. (2017) found that the conductive materials such as hematite and magnetite contained in the red mud could effectively enhance the activities of several key enzymes in the hydrolysis-acidification process, which triggered the electron transfer and the CO_2_ reduction to CH_4_ by methanogenesis in the following reaction (as shown in Eq. (6)):

6 $$ {\mathrm{CO}}_2+8{\mathrm{H}}^{+}+8{e}^{-}\to {\mathrm{CH}}_4+2{\mathrm{H}}_2\mathrm{O} $$

The results were consistent with the improved performance of methane production with the moderate dosage of nZVI-BC addition. Previous studies have reported that nZVI particles could increase the activity of methanogens, participate biosynthesis of the key enzymes, and optimize the structure of microbial community (Ren et al. 2007; Zhang et al. 2011). In addition, nZVI can also provide trace element which is necessary for the growth and metabolic process of microorganisms (Karri et al. 2010). From the community diversity analysis and combined with the methane yield in different treatment groups, it was clear that the addition of nZVI-BC changed the microbial structure. Furthermore, the result of the microbial analysis supports the mechanism assumption that nZVI could increase the relative abundance of *hydrogenotrophic* and reduce hydrogen pressure in AD system for improving the conversion of VFA (Kong et al. 2016).

## Conclusion

In this study, nanoscale zerovalent iron particles supported on corn stover-derived biochar (nZVI-BC) was synthesized, characterized, and used to investigate the impact of nZVI-BC on anaerobic digestion process at mesophilic temperature. It can be concluded that nZVI-BC addition could efficiently enhance methanogenic activity and increase the methane yield by 115.39%, but inhibitory effects were observed at high dosage. The composite of nZVI-BC also reduced free ammonia concentration, increased alkalinity, and improved the buffer capacity of the digestion system. All the nZVI-BC amended digesters shift the target heavy metals distribution from bioavailable fractions to the non-bioavailable fractions, which was at low potential ecological risk. Moreover, nZVI-BC evidently increased the diversity of archaea community and changed the methanogenic community structure. The results would provide the theoretical basis and technology supports for enhancing the stability of process and methane yields in sludge AD system.

## Abbreviations

AD: Anaerobic digestion
nZVI: Nanoscale zerovalent iron
nZVI-BC: Biochar supported nZVI
TS: Total solids
VS: Volatile solids
VFA: Volatile fatty acids
C/N: Carbon-to nitrogen ratio

## References

[CR1] Ahn HK, Smith MC, Kondrad SL, White JW (2010). Evaluation of biogas production potential by dry anaerobic digestion of switchgrass animal manure mixtures. Appl Biochem Biotechnol.

[CR2] Aikpokpodion PE, Lajide L, Aiyesanmi AF (2013) Characterization of heavy metal fractions in agricultural soils using sequential extraction technique. World J Agric Sci 6:51–57

[CR3] APHA (American Pubilc Health Association) (1998) Standards Methods for the Examination of Water and Wastewater. American Public Health Association, American Water Work Association, Water Environment federation, Washington DC, 252

[CR4] Appels L, Lauwers J, Degrève J, Helsen L, Lievens B, Willems K, Impe JV, Dewil R (2011). Anaerobic digestion in global bio-energy production: potential and research challenges. Renew Sust Energ Rev.

[CR5] Bagreev A, Bandosz TJ, Locke DC (2001). Pore structure and surface chemistry of adsorbents obtained by pyrolysis of sewage sludge-derived fertilizer. Carbon.

[CR6] Bakshi S, Banik C, Rathke SJ, Laird DA (2018). Arsenic sorption on zero-valent iron-biochar complexes. Water Res.

[CR7] Barker G (2011). Positive and negative carbon mineralization priming effects among a variety of biochar-amended soils. Soil Biol Biochem.

[CR8] Beesley L, Moreno-Jiménez E, Gomez-Eyles JL (2010). Effects of biochar and greenwaste compost amendments on mobility, bioavailability and toxicity of inorganic and organic contaminants in a multi-element polluted soil. Environ Pollut.

[CR9] Bruins MR, Kapil S, Oehme FW (2000). Microbial resistance to metals in the environment. Ecotoxicol Environ Saf.

[CR10] Chen Y, Cheng JJ, Creamer KS (2008). Inhibition of anaerobic digestion process: a review. Bioresour Technol.

[CR11] Chen Y, Yu B, Yin C, Zhang C, Dai X, Yuan H, Zhu N (2015). Biostimulation by direct voltage to enhance anaerobic digestion of waste activated sludge. RSC Adv.

[CR12] Chen Z, Cui X, Yan L, Zhang R, He Y, Wen W, Chang C, Liu G (2017). Maximization of the methane production from durian shell during anaerobic digestion. Bioresour Technol.

[CR13] Choong YY, Norli I, Abdullah AZ, Yhaya MF (2016). Impacts of trace element supplementation on the performance of anaerobic digestion process: a critical review. Bioresour Technol.

[CR14] Coutand M, Cyr M, Deydier E, Guilet R, Clastres P (2008). Characteristics of industrial and laboratory meat and bone meal ashes and their potential applications. J Hazard Mater.

[CR15] Cumbal L, Greenleaf J (2003). Polymer supported inorganic nanoparticles: characterization and environmental applications. React Funct Polym.

[CR16] Das KC, Garcia-Perez M, Bibens B, Melear N (2008). Slow pyrolysis of poultry litter and pine woody biomass: impact of chars and bio-oils on microbial growth. Environ Lett.

[CR17] Dearman B, Bentham RH (2007). Anaerobic digestion of food waste: comparing leachate exchange rates in sequential batch systems digesting food waste and biosolids. Waste Manag.

[CR18] Diao ZH, Du JJ, Jiang D, Kong LJ, Huo WY (2018). Insights into the simultaneous removal of Cr^6+^ and Pb^2+^ by a novel sewage sludge-derived biochar immobilized nanoscale zero valent iron: coexistence effect and mechanism. Sci Total Environ.

[CR19] Donatello S, Cheeseman CR (2013). Recycling and recovery routes for incinerated sewage sludge ash (ISSA): a review. Waste Manag.

[CR20] Dong B, Liu X, Dai L, Dai X (2013). Changes of heavy metal speciation during high-solid anaerobic digestion of sewage sludge. Bioresour Technol.

[CR21] Dong H, Deng J, Xie Y, Zhang C, Jiang Z, Cheng Y, Hou K, Zeng G (2017). Stabilization of nanoscale zero-valent iron (nZVI) with modified biochar for Cr (VI) removal from aqueous solution. J Hazard Mater.

[CR22] Eglal M, Ramamurthy A (2015). Removal of Pb (II), Cd (II), Cu (II) and trichloroethylene from water by Nanofer ZVI. Environ Lett.

[CR23] Engelhart M, Krüger M, Kopp J, Dichtl N (2000). Effects of disintegration on anaerobic degradation of sewage excess sludge in downflow stationary fixed film digesters. Water Sci Technol J Int Assoc Water Pollut Res.

[CR24] Fagbohungbe MO, Herbert BM, Hurst L, Ibeto CN, Li H, Usmani SQ, Semple KT (2016). The challenges of anaerobic digestion and the role of biochar in optimizing anaerobic digestion. Waste Manag.

[CR25] Fan L, Hao L, Guan D, Qi Y, Shao L, He P (2013). Synergetic stress of acids and ammonium on the shift in the methanogenic pathways during thermophilic anaerobic digestion of organics. Water Res.

[CR26] Feng Y, Zhang Y, Quan X, Chen S (2014). Enhanced anaerobic digestion of waste activated sludge digestion by the addition of zero valent iron. Water Res.

[CR27] Ganzoury MA, Allam NK (2015). Impact of nanotechnology on biogas production: a mini-review. Renew Sust Energ Rev.

[CR28] Garcia JL, Patel BK, Ollivier B (2000). Taxonomic, phylogenetic, and ecological diversity of methanogenic archaea. Anaerobe.

[CR29] Gong X, Huang D, Liu Y, Zeng G, Wang R, Wan J, Zhang C, Cheng M, Qin X, Xue W (2017). Stabilized nanoscale zero-valent iron mediated cadmium accumulation and oxidative damage of Boehmeria nivea (L.) Gaudich cultivated in cadmium contaminated sediments. Environ Sci Technol.

[CR30] Hashimoto AG (1986). Ammonia inhibition of methanogenesis from cattle wastes. Agric Wastes.

[CR31] He F, Zhao D, Paul C (2010). Field assessment of carboxymethyl cellulose stabilized iron nanoparticles for in situ destruction of chlorinated solvents in source zones. Water Res.

[CR32] He R, Peng Z, Lyu H, Huang H, Nan Q, Tang J (2018). Synthesis and characterization of an iron-impregnated biochar for aqueous arsenic removal. Sci Total Environ.

[CR33] Huang H, He L, Lei Z, Zhang Z (2015). Contribution of precipitates formed in fermentation liquor to the enhanced biogasification of ammonia-rich swine manure by wheat-rice-stone addition. Bioresour Technol.

[CR34] Jabeen H, Kemp KC, Chandra V (2013). Synthesis of nano zerovalent iron nanoparticles--graphene composite for the treatment of lead contaminated water. J Environ Manag.

[CR35] Jiang S, Park S, Yoon Y, Lee JH, Wu WM, Phuoc DN, Sadowsky MJ, Hur HG (2013). Methanogenesis facilitated by geobiochemical iron cycle in a novel syntrophic methanogenic microbial community. Environ Sci Technol.

[CR36] Karri S, Sierra-Alvarez R, Field JA (2010). Zero valent iron as an electron-donor for methanogenesis and sulfate reduction in anaerobic sludge. Biotechnol Bioeng.

[CR37] Keane E (2010) Fate, transport and toxicity of nanoscale zero-valent Iron (nZVI) used during superfund remediation. US Environmental Protection Agency 69(11):2357–2363

[CR38] Khan S, Chao C, Waqas M, Arp HPH, Zhu YG (2013). Sewage sludge biochar influence upon rice (Oryza sativa L) yield, metal bioaccumulation and greenhouse gas emissions from acidic Paddy soil. Environ Sci Technol.

[CR39] Koch K, Fernández YB, Drewes JE (2015). Influence of headspace flushing on methane production in biochemical methane potential (BMP) tests. Bioresour Technol.

[CR40] Kong X, Wei Y, Xu S, Liu J, Li H, Liu Y, Yu S (2016). Inhibiting excessive acidification using zero-valent iron in anaerobic digestion of food waste at high organic load rates. Bioresour Technol.

[CR41] Kong X, Yu S, Xu S, Fang W, Liu J, Li H (2018). Effect of Fe0 addition on volatile fatty acids evolution on anaerobic digestion at high organic loading rates. Waste Manag.

[CR42] Li XQ, Brown DG, Zhang WX (2007). Stabilization of biosolids with nanoscale zero-valent iron (nZVI). J Nanopart Res.

[CR43] Liu Y, Zhang Y, Quan X, Li Y, Zhao Z, Meng X, Chen S (2012). Optimization of anaerobic acidogenesis by adding Fe 0 powder to enhance anaerobic wastewater treatment. Chem Eng J.

[CR44] Liu C, Li H, Zhang Y, Si D, Chen Q (2016). Evolution of microbial community along with increasing solid concentration during high-solids anaerobic digestion of sewage sludge. Bioresour Technol.

[CR45] Luo C, Lü F, Shao L, He P (2015). Application of eco-compatible biochar in anaerobic digestion to relieve acid stress and promote the selective colonization of functional microbes. Water Res.

[CR46] Madsen M, Holm-Nielsen JB, Esbensen KH (2011). Monitoring of anaerobic digestion processes: a review perspective. Renew Sust Energ Rev.

[CR47] Mao CL, Feng YZ, Wang XJ, Ren GX (2015). Review on research achievements of biogas from anaerobic digestion. Renew Sust Energ Rev.

[CR48] Maroušek J, Vochozka M, Plachý J, Žák J (2017). Glory and misery of biochar. Clean Technol Environ.

[CR49] Meng X, Zhang Y, Li Q, Quan X (2013). Adding Fe^0^ powder to enhance the anaerobic conversion of propionate to acetate. Biochem Eng J.

[CR50] Mohan D, Sarswat A, Ok YS, Jr PC (2014). Organic and inorganic contaminants removal from water with biochar, a renewable, low cost and sustainable adsorbent—a critical review. Bioresour Technol.

[CR51] Mumme J, Srocke F, Heeg K, Werner M (2014). Use of biochars in anaerobic digestion. Bioresour Technol.

[CR52] Neeli S, Ramsurn H (2018). Synthesis and formation mechanism of iron nanoparticles in graphitized carbon matrices using biochar from biomass model compounds as a support. Carbon.

[CR53] Puga AP, Abreu CA, Lca M, Beesley L (2015). Biochar application to a contaminated soil reduces the availability and plant uptake of zinc, lead and cadmium. J Environ Manag.

[CR54] Qian L, Shang X, Zhang B, Zhang W, Su A, Ch Y, O D, H L, Y J, Ch M (2019). Enhanced removal of Cr (VI) by silicon rich biochar-supported nanoscale zero-valent iron. Chemosphere.

[CR55] Qiu X, Fang Z, Liang B, Gu F, Xu Z (2011). Degradation of decabromodiphenyl ether by nano zero-valent iron immobilized in mesoporous silica microspheres. J Hazard Mater.

[CR56] Rajapaksha AU, Chen SS, Tsang DC, Zhang M, Vithanage M, Mandal S, Gao B, Bolan NS, Ok YS (2016). Engineered/designer biochar for contaminant removal/immobilization from soil and water: potential and implication of biochar modification. Chemosphere.

[CR57] Ren NQ, Chua HS, Chan Y, Tsang YF, Wang YJ, Sin N (2007). Assessing optimal fermentation type for bio-hydrogen production in continuous-flow acidogenic reactors. Bioresour Technol.

[CR58] Shang J, Zong M, Yu Y, Kong X, Du Q, Liao Q (2017). Removal of chromium (VI) from water using nanoscale zerovalent iron particles supported on herb-residue biochar. J Environ Manag.

[CR59] Shen Y, Linville JL, Leon ID, Schoene RP, Urgun-Demirtas M (2016). Towards a sustainable paradigm of waste-to-energy process: enhanced anerobic digestion of sludge with woody biochar. J Clean Prod.

[CR60] Shen Z, Zhang J, Hou D, Tsang D, Ok Y, Alessi D (2019) Synthesis of MgO-coated corncob biochar and its application in lead stabilization in a soil washing residue. Environ Int 122:357–362 10.1016/j.envint.2018.11.045 30501914

[CR61] Shi J, Xu F, Wang Z, Stiverson JA, Yu Z, Li Y (2014) Effects of microbial and non-microbial factors of liquid anaerobic digestion effluent as inoculum on solid-state anaerobic digestion of corn stover. Bioresource Technol 157:188–196 10.1016/j.biortech.2014.01.089 24556372

[CR62] Shu D, He Y, Yue H, Wang Q (2015). Microbial structures and community functions of anaerobic sludge in six full-scale wastewater treatment plants as revealed by 454 high-throughput pyrosequencing. Bioresour Technol.

[CR63] Sizmur T, Fresno T, Akgül G, Frost H, Morenojiménez E (2017). Biochar modification to enhance sorption of inorganics from water. Bioresour Technol.

[CR64] Su L, Guo G, Zhao A, Zhao Y (2013). Stabilization of sewage sludge in the presence of nanoscale zero-valent iron (nZVI): abatement of odor and improvement of biogas production. J Mater Cycles Waste.

[CR65] Su C, Li W, Lu Y, Chen M, Huang Z (2016). Effect of heterogeneous Fenton-like pre-treatment on anaerobic granular sludge performance and microbial community for the treatment of traditional Chinese medicine wastewater. J Hazard Mater.

[CR66] Su H, Fang Z, Tsang PE, Fang J, Zhao D (2016). Stabilisation of nanoscale zero-valent iron with biochar for enhanced transport and in-situ remediation of hexavalent chromium in soil. Environ Pollut.

[CR67] Su H, Fang Z, Tsang PE, Zheng L, Cheng W, Fang J, Zhao D (2016). Remediation of hexavalent chromium contaminated soil by biochar-supported zero-valent iron nanoparticles. J Hazard Mater.

[CR68] Suanon F, Sun Q, Li M, Cai X, Zhang Y, Yan Y, Yu C (2017). Application of nanoscale zero valent iron and iron powder duringsludge anaerobic digestion: impact on methane yield andpharmaceutical and personal care products degradation. J Hazard Mater.

[CR69] T P, N S, K S, RD T, GV L (2007). Aggregation and sedimentation of aqueous nanoscale zerovalent iron dispersions. Environ Sci Technol.

[CR70] Tian Q, Wang Q, Xie Q, Li J (2010). Aqueous solution preparation, structure, and magnetic properties of Nano-granular ZnxFe_3−_xO_4_Ferrite films. Nanoscale Res Lett.

[CR71] Tian X, Wang W, Tian N, Zhou C, Yang C, Komarneni S (2016). Cr (VI) reduction and immobilization by novel carbonaceous modified magnetic Fe_3_O_4_/halloysite nanohybrid. J Hazard Mater.

[CR72] Torri C, Fabbri D (2014). Biochar enables anaerobic digestion of aqueous phase from intermediate pyrolysis of biomass. Bioresour Technol.

[CR73] Vrieze JD, Hennebel T, Boon N, Verstraete W (2012). Methanosarcina: the rediscovered methanogen for heavy duty biomethanation. Bioresour Technol.

[CR74] Wang W, Han H (2012). Recovery strategies for tackling the impact of phenolic compounds in a UASB reactor treating coal gasification wastewater. Bioresour Technol.

[CR75] Wang Y, Shi J, Wang H, Lin Q, Chen X, Chen Y (2007). The influence of soil heavy metals pollution on soil microbial biomass, enzyme activity, and community composition near a copper smelter. Ecotoxicol Environ Saf.

[CR76] Wang FH, Zhang F, Chen YJ, Gao J, Zhao B (2015). A comparative study on the heavy metal solidification/stabilization performance of four chemical solidifying agents in municipal solid waste incineration fly ash. J Hazard Mater.

[CR77] Wang S, Gao B, Zimmerman AR, Li Y, Ma L, Harris WG, Migliaccio KW (2015). Physicochemical and sorptive properties of biochars derived from woody and herbaceous biomass. Chemosphere.

[CR78] Wang S, Gao B, Li Y, Creamer AE, He F (2017). Adsorptive removal of arsenate from aqueous solutions by biochar supported zero-valent iron nanocomposite: batch and continuous flow tests. J Hazard Mater.

[CR79] Wang YC, Shen JK, Xiang WN (2018). Ecosystem service of green infrastructure for adaptation to urban growth: function and configuration. Ecosyst Health Suatain.

[CR80] Watling HR, Collinson DM, Shiers DW, Bryan CG, Watkin ELJ (2013). Effects of pH, temperature and solids loading on microbial community structure during batch culture on a polymetallic ore. Miner Eng.

[CR81] Wei AL, Ma J, Chen JJ, Zhang Y, Song JX, Yu XY (2018). Enhanced nitrate removal and high selectivity towards dinitrogen for groundwater remediation using biochar-supported nano zero-valent iron. Chem Eng J.

[CR82] Wei J, Hao XD, Loosdrecht CM, Li J (2018). Feasibility analysis of anaerobic digestion of excess sludge enhanced by iron: a review. Renew Sust Energ Rev.

[CR83] Xi Y, Chang Z, Ye X, Xu R, Du J, Chen G (2014). Methane production from wheat straw with anaerobic sludge by heme supplementation. Bioresour Technol.

[CR84] Xie Y, Dong H, Zeng G, Tang L, Jiang Z, Zhang C, Deng J, Li Z, Zhang Y (2017). The interactions between nanoscale zero-valent iron and microbes in the subsurface environment: A review. J Hazard Mater.

[CR85] Yang Y, Guo J, Hu Z (2013). Impact of nano zero valent iron (NZVI) on methanogenic activity and population dynamics in anaerobic digestion. Water Res.

[CR86] Yang F, Zhang S, Sun Y, Cheng K, Li J, Tsang DCW (2018) Fabrication and characterization of hydrophilic corn stalk biochar-supported nanoscale zero-valent iron composites for efficient metal removal. Bioresource Technol 265:490–497 10.1016/j.biortech.2018.06.029 29940499

[CR87] Ye J, Hu A, Ren G, Zhou T, Zhang G, Zhou S (2017). Red mud enhances methanogenesis with the simultaneous improvement of hydrolysis-acidification and electrical conductivity. Bioresour Technol.

[CR88] Ying M, Sang L, Hairong Y, Dexun Z, Yanping L, Baoning Z, Akiber C, Muhammad J, Xiujin L (2015). Evaluating biomethane production from anaerobic mono- and co-digestion of food waste and floatable oil (FO) skimmed from food waste. Bioresour Technol.

[CR89] Yoon IH, Bang S, Chang JS, Min GK, Kim KW (2011). Effects of pH and dissolved oxygen on Cr (VI) removal in Fe (0)/H_2_O systems. J Hazard Mater.

[CR90] Yuan H, Chen Y, Dai X, Zhu N (2016). Kinetics and microbial community analysis of sludge anaerobic digestion based on micro-direct current treatment under different initial pH values. Energy.

[CR91] Zhang Y, Jing Y, Quan X, Liu Y, Onu P (2011). A built-in zero valent iron anaerobic reactor to enhance treatment of azo dye wastewater. Water Sci Technol.

[CR92] Zhang Y, Feng Y, Quan X (2015). Zero-valent iron enhanced methanogenic activity in anaerobic digestion of waste activated sludge after heat and alkali pretreatment. Waste Manag.

[CR93] Zhang M, Yang C, Jing Y, Li J (2016). Effect of energy grass on methane production and heavy metal fractionation during anaerobic digestion of sewage sludge. Waste Manag.

[CR94] Zhang Z, Gao P, Cheng J, Liu G, Zhang X, Feng Y (2018). Enhancing anaerobic digestion and methane production of tetracycline wastewater in EGSB reactor with GAC/NZVI mediator. Water Res.

[CR95] Zhao P, Shen Y, Ge S, Chen Z, Yoshikawa K (2016). ChemInform abstract: clean solid biofuel production from high moisture content waste biomass employing hydrothermal treatment. Appl Energy.

[CR96] Zhou Y, Gao B, Zimmerman AR, Chen H, Zhang M, Cao X (2014). Biochar-supported zerovalent iron for removal of various contaminants from aqueous solutions. Bioresour Technol.

[CR97] Zhu L, Yin S, Yin Q, Wang H (2015) Biochar: a new promising catalyst support using methanation as a probe reaction. Energy Sci Eng 3(2):126–134

[CR98] Zhu L, Tong L, Zhao N, Li J, Lv Y (2019). Coupling interaction between porous biochar and nano zero valent iron/nano α-hydroxyl iron oxide improves the remediation efficiency of cadmium in aqueous solution. Chemosphere.

[CR99] Zwieten LV, Kimber S, Morris S, Chan KY, Downie A, Rust J, Joseph S, Cowie A (2010). Effects of biochar from slow pyrolysis of papermill waste on agronomic performance and soil fertility. Plant Soil.

